# Three-dimensional microsurgical anatomy of the cerebral hemisphere from medial to lateral: a fiber-dissection study

**DOI:** 10.1007/s00701-026-06829-z

**Published:** 2026-03-08

**Authors:** Chen Li, Wenqi Zhong, Haibo Li, Yinan Tao, Jiaoyan Huang, Junfeng Lu, Xiaoluo Zhang, Jinsong Wu

**Affiliations:** 1 Department of Neurosurgery, Huashan Hospital, Shanghai Medical College, Fudan University, No. 12 Middle Wulumuqi Rd., Shanghai, China; 2 Shanghai Key Laboratory of Brain Function Restoration and Neural Regeneration, Fudan University, Shanghai, China; 3 Brain Function Laboratory, Neurosurgical Institute of Fudan University, Shanghai, China; 4 Department of Neurosurgery, Anhui Digital Brain Engineering Research Center & The First Affiliated Hospital of Wannan Medical College, Yijishan Hospital of Wannan Medical College, Wuhu, China; 5 Maanshan People’s Hospital, Maanshan, China; 6 Human Anatomy Experimental Training Center, School of Basic Medical Sciences, Wannan Medical College, Wuhu, China

**Keywords:** Three-dimensional, Medial surface, Cerebrum, Fiber dissection, Microsurgical anatomy

## Abstract

**Background:**

Accurate exposure of lesions on the medial cerebral hemisphere remains technically challenging, and current imaging cannot fully depict the subcortical intricate architecture extending from the medial surface outward. Although portions of this anatomy have been described, a comprehensive topographic characterization from medial to lateral is still lacking.

**Objectives:**

To provide a systematic, layer-by-layer topographic analysis of the white-matter fiber tracts and deep gray-matter nuclei from the medial surface to the lateral convexity of the cerebral hemisphere by combining stepwise fiber dissection with three-dimensional (3D) photography.

**Methods:**

Twelve adult human cerebral hemispheres, fixed in 10% formalin and prepared with the Klingler fiber-dissection technique, were examined under 6× – 40 × magnification. Dissection commenced at the medial surface and proceeded outward, exposing commissural, association, and projection fibers as well as adjacent subcortical nuclei. High-resolution stereoscopic images were captured after each stage to document 3D spatial relationships.

**Results:**

From medial to lateral, the hemisphere comprised orderly layers of commissural, association, and projection systems interwoven with deep nuclei, forming a complex but reproducible arrangement in all specimens. The study provides complete medial exposure of these structures and demonstrates consistent positional relationships among different specimens.

**Conclusions:**

This 3D fiber-dissection study offers the layer-by-layer depiction of the cerebral hemisphere from medial to lateral, clarifying spatial relationships among key white-matter bundles and deep nuclei. The anatomic insights gained may facilitate safer, more precise neurosurgical approaches and refine understanding of hemispheric connectivity.

**Supplementary Information:**

The online version contains supplementary material available at 10.1007/s00701-026-06829-z.

## Introduction

Designing safe and minimally invasive surgical approaches to lesions within the cerebral hemisphere remains a primary objective for neurosurgeons, with corresponding microsurgical anatomical research serving as an essential foundation [29, 49]. Prior microsurgical and imaging literature richly describes brain anatomy; however, there are relatively few systematic, comprehensive, and fine-grained studies that follow the hemisphere from its medial surface all the way to the lateral surface [2, 32, 35, 41, 42, 47]. Compared with lesions located laterally, medial hemispheric lesions pose significant surgical challenges, as inadvertent injury to adjacent anatomical structures frequently results in unexpected complications [35, 50]. Medial approaches are also surgical challenging due to other factors, as bridging veins along the interhemispheric fissure. The complex interrelationships between medial white-matter fiber tracts and subcortical gray-matter nuclei remain incompletely delineated. Despite major advances in diffusion MRI and tractography, radiological reconstruction still faces intrinsic constraints when applied to the deep midline and periventricular regions. The spatial resolution of clinical diffusion imaging remains coarse relative to the millimeter-scale laminar organization of commissural, association, and projection fibers, leading to partial-volume effects that are accentuated near the ventricles and along the corpus callosum. In addition, densely packed crossing, kissing, and fanning fibers within the central core may be inadequately modeled by standard tractography assumptions, which can reduce reliability in disentangling closely apposed bundles and in depicting stepwise ‘layer-by-layer’ architecture. These limitations are particularly relevant when interpreting medial hemispheric pathways that are interwoven with deep nuclei and periventricular fiber systems [17, 36, 41].

In the present study, we combined the fiber dissection technique with three-dimensional (3D) photography, systematically dissecting the cerebral hemisphere layer by layer, starting from the medial surface and progressing outward to superficial lateral association fibers. This approach allowed a comprehensive and detailed microsurgical anatomical analysis of the cortical surface, white matter fiber tracts, and subcortical nuclei from medial to lateral.

## Methods

Twelve human cerebral hemispheres were harvested from cadavers and fixed in 10% formalin. After fixation, arachnoid mater, pia mater, and superficial vessels were removed from the cortical surface under microscopic visualization. Following the Klingler method, specimens were frozen at −16 °C for two weeks [8, 13, 18, 19]. This freezing step induced water expansion and ice crystal formation within the brain tissue, facilitating the separation and visualization of main white matter fibers during dissection. Subsequently, specimens were thawed at room temperature and dissected layer by layer under 6 × –40 × magnification using a Zeiss surgical microscope. Throughout dissection, specimens were intermittently preserved in 70% alcohol. Dissection began from the medial cerebral surface and progressed laterally, systematically exposing fiber bundles layer by layer. Each dissection stage was documented using three-dimensional (3D) photography to generate stereoscopic images. Each 3D image was accompanied by a corresponding annotated two-dimensional (2D) image. Stereoscopic images were processed using Adobe Photoshop CC 2023 (Adobe, San Jose, CA, USA), version 12.0 X 64-bit, and viewed with red-blue 3D glasses. All photographs were captured using a professional digital camera (Canon EOS 5D Mark IV), equipped with a 100-mm macro lens (Canon) and ring flash (Canon). Twelve cerebral hemispheres were examined to assess the reproducibility of the stepwise medial-to-lateral dissection sequence across specimens; in all hemispheres, the principal spatial relationships among commissural, association, and projection systems and the adjacent deep nuclei were consistent.

The specimens were obtained through the Anhui Red Cross body donation receiving station, a legally authorized institution for accepting voluntarily donated human remains. At the time of donation, the receiving station informed the donors and their families that the donated bodies would be used for medical education and scientific research. All specimens were handled in accordance with local regulations and under institutional ethical oversight.

Illustrative Surgical Case: This study includes one representative surgical case to demonstrate the potential translational application of the medial-to-lateral anatomical framework in surgery. The surgical approach was determined based on comprehensive preoperative anatomical evaluation, diffusion tensor imaging (DTI) findings, and guidance from intraoperative neurophysiological monitoring. This study was conducted in accordance with institutional regulations, and appropriate ethical approval and patient informed consent were obtained.

## Results

**Fig. 1:**
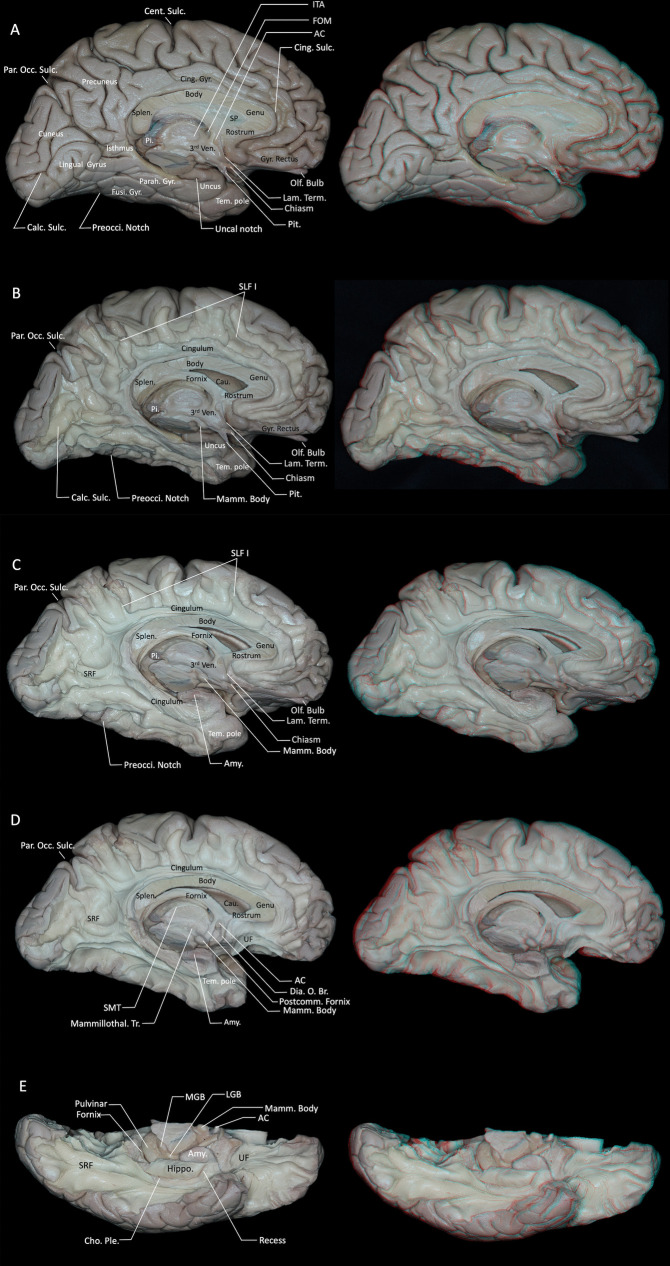
**A** Medial view of the cerebrum, highlighting its complexity. **B** Following removal of the cortical gray matter, the first layer of U-shaped fibers was exposed. **C** Subsequent removal of the U-shaped fibers exposed superficial association fibers arranged from superior to inferior, including the SLF I, cingulum, corpus callosum, fornix, and SRF. The SRF follows a dorsomedial-to-ventrolateral path, linking the anterior portions of the cuneus and lingual gyrus with the isthmus of the cingulate gyrus and the posterior parahippocampal region. The cingulum originating from the paraterminal and paraolfactory gyri, coursing above the corpus callosum, and terminating in the amygdala. **D** The cingulum within the parahippocampal gyrus and the medial portion of the hippocampus were dissected to expose the fimbria of the hippocampus, the crura of the fornix, and the amygdala. **E** Basal view, after removing the cingulum, the hippocampus is exposed; anterior to the hippocampal head lies a recess. AC = anterior commissure; Amy. = amygdala; Calc. = calcarine; Cau. = caudate nucleus; Cent. = central; Cho. = choroid; Cing. = cingulate; Dia. O. Br. = diagonal band of Broca; FOM = foramen of Monro; Fusi. = fusiform; Gyr. = gyrus; Hippo. = hippocampus; ITA = interthalamic adhesion; Lam. = lamina; Mamm. = mammillary; LGB = lateral geniculate body; Mammillothal. = mammillothalamic; MGB = medial geniculate body; Occ. = occipital; Olf. = olfactory; Par. = parieto; Parah. = parahippocampal; Pi. = pineal; Pit. = pituitary stalk; Ple. = plexus; Postcomm. = postcommissural; Preocci. = preoccipital; SLF = superior longitudinal fasciculus; SP = septum pellucidum; Splen. = splenium; SMT = stria medullaris thalami; SRF = sledge runner fasciculus; Sulc. = sulcus; Tem. = temporal; Term. = terminalis; Tr. = tracts; UF = uncinate fasciculus; Ven. = ventricle. Right-hand panels show 3D reconstructions of the corresponding dissection stages

Anatomical landmarks on the medial surface of the cerebral hemisphere were systematically identified. The medial surface includes the medial aspects of the frontal, parietal, occipital, and temporal lobes as well as structures of the limbic lobe [29, 30]. On the medial frontal surface, the medial part of the superior frontal gyrus and the anterior part of the paracentral lobule are located superior to the anterior cingulate gyrus, which is classically considered part of the limbic lobe. The medial parietal region includes the precuneus, the posterior part of the paracentral lobule, and the posterior cingulate gyrus. The medial occipital region is composed of the cuneus superiorly and the lingual gyrus inferiorly, separated by the calcarine sulcus. The medial temporal region is formed by the parahippocampal gyrus, whose anterior portion constitutes the uncus. Unlike the convex lateral surface and the concave basal surface, the medial cerebral surface is relatively flat (Fig. 1A).

Following removal of the cortical gray matter, the first layer of U-shaped fibers was exposed. Removal of the septum pellucidum exposed the anterior horn and body of the lateral ventricle. (Fig. 1B). Subsequent removal of the U-shaped fibers exposed a superficial layer of long white matter tracts arranged from superior to inferior, including the association fibers of the superior longitudinal fasciculus (SLF) I, the cingulum, and the sledge runner fasciculus (SRF), as well as the commissural fibers of the corpus callosum and the projection fibers of the fornix. (Fig. 1B, C). The SLF I is located within the superior bank of the cingulate sulcus, precuneus, and medial surface of the superior frontal gyrus, running superior to the cingulum and medial to the corpus callosum fibers [21]. However, the precise composition and even the existence of a distinct SLF I remains a matter of debate, and some authors have proposed that the dorsal fronto-parietal connections in this region may instead represent a collection of short U-shaped fibers rather than a single long association bundle [21, 46]. The sledge runner fasciculus (SRF) courses obliquely beneath the U-fibers of the medial occipital lobe, with a trajectory that is nearly perpendicular to them. It follows a dorsomedial-to-ventrolateral path, linking the anterior portions of the cuneus and lingual gyrus with the isthmus of the cingulate gyrus and the posterior parahippocampal region [2].

Removal of the cortical gray matter of the cingulate gyrus revealed the cingulum, which originating from the paraterminal and parolfactory gyri, coursing above the corpus callosum, and terminating in the amygdala (Fig. 1C). During fiber dissection, connections between the cingulum and the anterior ventral aspect of the amygdala were identified (Fig. 1C, D). Fibers originating from the medial frontal lobe, paracentral lobule, precuneus, and cuneus merge anteriorly into the cingulum, making the tract less prominent medially in the frontal lobe but wider as it progresses toward the medial surfaces of the parietal and occipital lobes. The segment of the cingulum originating from the amygdala and hippocampus represents the longest fiber tracts and is located most medially. Apart from medial connections, the cingulum is bordered superiorly, laterally, and inferiorly by cortical gray matter (Fig. 1D).

Beneath the cingulate gyrus and above the corpus callosum is situated a structure known as the indusium griseum (IG), or supracallosal gyrus. Anatomical observation revealed that the IG is a slender, ribbon-shaped structure averaging around 2 cm in width, with a range between 1 and 3.4 cm [7, 44]. It runs along the dorsal surface of the corpus callosum, extending anteriorly over the lamina terminalis toward the septal region and posteriorly attaching to the splenium of the corpus callosum, becoming narrower at both ends [7, 44]. Consistent with classical descriptions, the IG can be regarded as a dorsal continuation of the hippocampal formation.

**Fig. 2:**
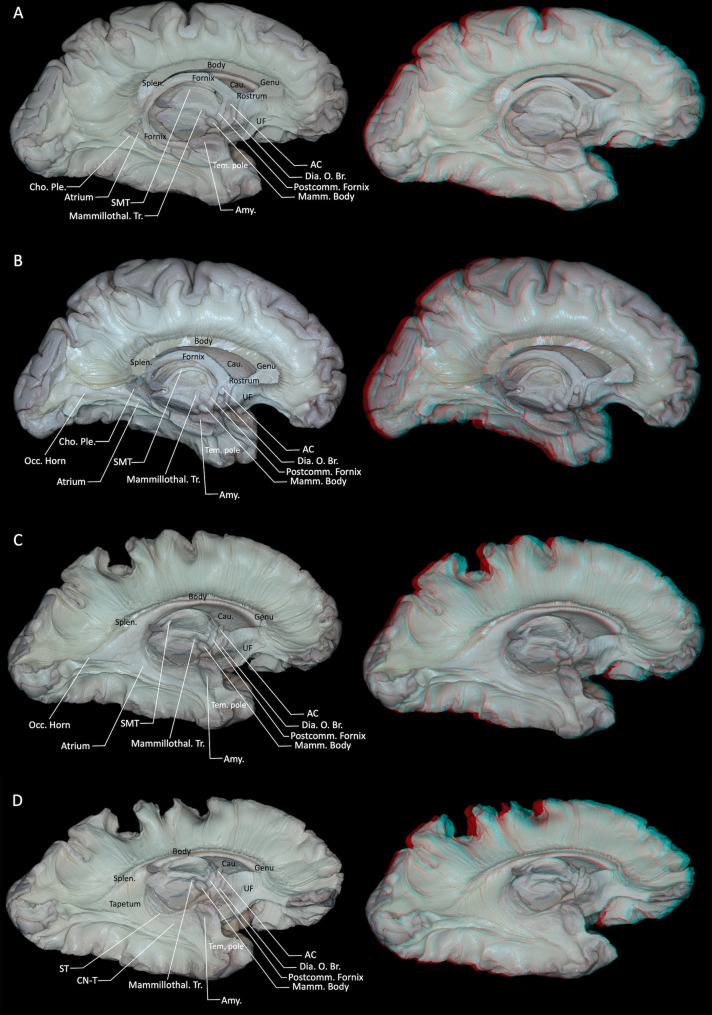
**A** Further dissection of the hippocampal body and tail reveals the atrium of the lateral ventricle and the choroid plexus. **B** Lateral view of deep midline structures including the corpus callosum, fornix, pineal gland, and stria medullaris thalami. **C** Removal of the hippocampus, fimbria, and crura of the fornix exposes the full extent of the atrium of the lateral ventricle. **D** Following ependymal removal, sequential medial-to-lateral exposure reveals the stria terminalis, the tail of the caudate nucleus, and the tapetum. Callosal fibers projecting superiorly into the lateral ventricle were selectively removed, leaving behind fibers that could not be further dissected in the superior direction. AC = anterior commissure; Amy. = amygdala; Cau. = caudate nucleus; Cho. = choroid; CN-T = tail of the caudate nucleus; Dia. O. Br. = diagonal band of Broca; Mamm. = mammillary; Mammillothal. = mammillothalamic; Occ. = occipital; Ple. = plexus; Postcomm. = postcommissural; Splen. = splenium; SMT = stria medullaris thalami; ST = stria terminalis; Tem. = temporal; Tr. = tracts; UF = uncinate fasciculus. Right-hand panels show 3D reconstructions of the corresponding dissection stages

**Fig. 3:**
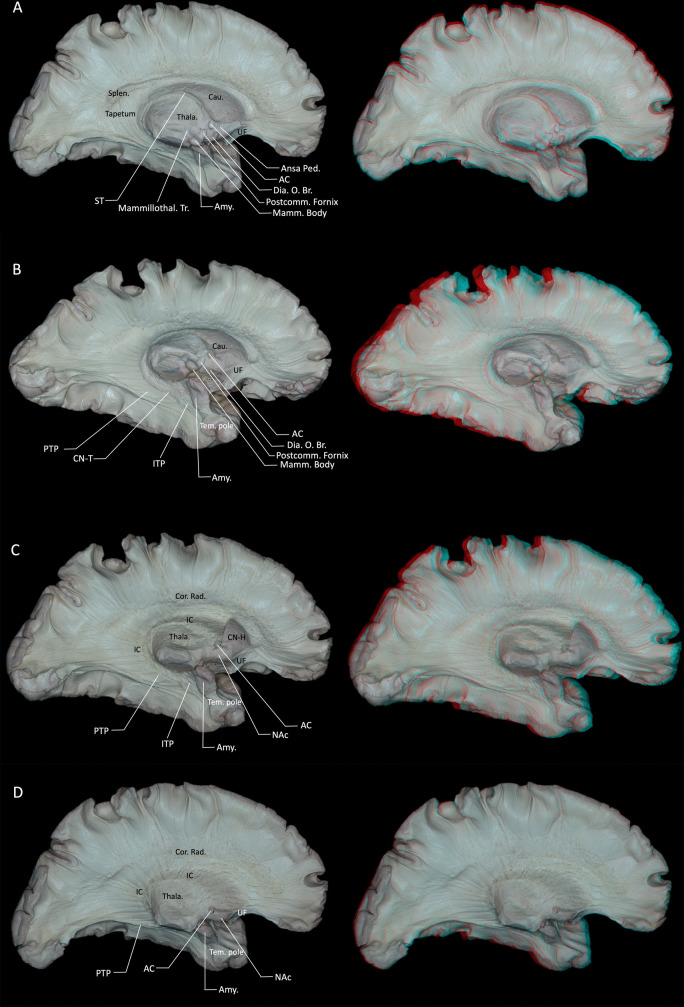
**A** Exposure of the caudate nucleus followed by dissection of the overlying corpus callosum revealed that fibers beneath the callosum descend in a downward trajectory. **B** After removal of the left stria terminalis and the caudate tail, the sublentiform part of the internal capsule (IC-SL) becomes visible. This region contains both the ITP and the PTP. The ITP carries fibers linking the thalamic pulvinar to the amygdala and temporal pole, whereas the PTP is composed of reciprocal thalamocortical and corticothalamic fibers connecting the pulvinar to the temporal and occipital lobes, including components of the optic and auditory radiations. **C** The head of the caudate nucleus was preserved while the remaining caudate structure was dissected. The internal and external capsules converge superior to the putamen to form the corona radiata. **D** Removal of the caudate head exposed the underlying nucleus accumbens. AC = anterior commissure; Amy. = amygdala; Cau. = caudate nucleus; CN-H = Head of the caudate nucleus; CN-T = tail of the caudate nucleus; Cor. = corona; Dia. O. Br. = diagonal band of Broca; IC = internal capsule; ITP = inferior thalamic peduncle; Mamm. = mammillary; Mammillothal. = mammillothalamic; NAc = nucleus accumbens; Ped. = peduncularis; Postcomm. = postcommissural; PTP = posterior thalamic peduncle; Rad. = radiata; SMT = stria medullaris thalami; ST = stria terminalis; Tem. = temporal; Thala. = thalamus; Tr. = tracts. Right-hand panels show 3D reconstructions of the corresponding dissection stages

Following the removal of the cingulum and indusium griseum, the corpus callosum beneath was clearly visible. Viewed from the medial aspect, the corpus callosum appeared as a distinct C-shaped arching above the lateral ventricle, typically divided into four segments: the rostrum, genu, body, and splenium (Fig. 1D). Fibers of the corpus callosum had an intimate relationship with the lateral ventricles, forming several of its walls and influencing associated anatomical and functional areas. Specifically, fibers from the callosal body created the superior boundary of the ventricular body; fibers from the rostrum contributed to the inferior wall of the frontal horn; fibers of the genu (forceps minor) entered the frontal lobes, forming the anteromedial boundary of the frontal horn; and fibers from the splenium (forceps major) entered the occipital lobes, delineating the medial wall of the occipital horn and creating the characteristic enlargement. Inferiorly, the tapetal fibers originated from the body and splenium of the corpus callosum and coursed lateral to the atrium, encircling the occipital horn, atrium (trigone), and temporal horn of the lateral ventricle, where they formed parts of the lateral and superior walls. In this region, the tapetum also contributes to the composition of the sagittal stratum [9, 22]. (Fig. 2B, C). Dissection of the corpus callosum initially exposed fibers extending upward to the top of the frontal-parietal regions. However, upon further dissection, approximately 2 mm thick fibers were found to project downward instead. Fibers from the splenium extended downward forming the tapetum, while fibers from the body, genu, and rostrum descended medially to merge with the internal capsule (Fig. 3A). Initial upward dissection of callosal fibers subsequently exposed their downward trajectory. It should be emphasized, however, that callosal fibers fan out to almost the entire cortical mantle and are intricately interwoven with long association and projection pathways. As a consequence, the fiber dissection technique can only follow a subset of all callosal fibers, and more peripheral commissural fibers are likely to be disrupted or lost during the dissection process.

Located inferiorly to the corpus callosum, the fornix surrounded the thalamus (Fig. 2A). The hippocampal fimbria primarily comprised fibers arising from the hippocampal formation. In our dissections, we also observed a subset of fibers that appeared to converge onto the fimbria from the region of the basolateral amygdala, suggesting a possible anatomical substrate for amygdalo-hippocampal projections described in experimental tract-tracing studies in non-human primates and rodents [24]. These fibers converged medially, ascending to form the right and left crura of the fornix (Fig. 2A). As they reached the midline, the two crura became the bodies of the fornix, which coursed in close apposition and traveled horizontally forward beneath the corpus callosum while largely remaining two independent bundles. Anteriorly, the fornix body sharply curved downward, forming the columns of the fornix, which defined the superior and anterior boundaries of the foramen of Monro (Fig. 2A). At the level of the anterior commissure, the columns bifurcated into precommissural and postcommissural segments. The postcommissural fibers traveled posteriorly to the anterior commissure, terminating in the mammillary bodies of the hypothalamus, whereas the precommissural fibers coursed anteriorly toward the septal region and primarily terminated in the septal nuclei, with a smaller contingent projecting to the posterosuperior portion of the nucleus accumbens near the septal area (Fig. 1D) [1, 40]. Additionally, the commissure of the fornix was located at the crura, containing a few fibers connecting the bilateral hippocampi. Following dissection of the body of the fornix, the deeper structures—the stria medullaris thalami and stria terminalis—became visible (Fig. 2A, D). Our dissections revealed that the stria medullaris thalami lie along the dorsal margin of the third ventricle, medial to the thalamus. Anteriorly, it connects basal frontal structures associated with the limbic system, including the anterior cingulate cortex, orbitofrontal cortex, septal area, and anterior olfactory nucleus (Fig. 2A) [26]. Additionally, it is connected posteriorly with the habenula and pineal gland.

The stria terminalis represents a significant limbic fiber pathway connecting the amygdala with hypothalamic and thalamic midline nuclei. It originates from the central nucleus of the amygdala, ascends along the inferior horn of the lateral ventricle, courses between the caudate nucleus and thalamus, and terminates near the anterior commissure, projecting towards the preoptic region, paraventricular nucleus, periaqueductal gray matter, and the bed nucleus of the stria terminalis, situated close to the anterior commissure (Fig. 2B, D) [1]. Anatomically, the stria terminalis closely follows the medial border of the caudate nucleus and the dorsolateral border of the thalamus, curving around the body and anterior horn of the lateral ventricle. The space where the stria terminalis is placed is called striothalamic sulcus. Subsequent removal of the hippocampal head, fornix, stria medullaris thalami, choroid plexus, and superior corpus callosum revealed the medial aspect of the lateral ventricle, with the caudate tail slightly protruding into the ependymal surface (Fig. 2D).

After fully dissecting the posterior segment of the corpus callosum, including the tapetum, the posterior limb of the internal capsule was exposed. The internal capsule is an extensive collection of projection fibers positioned between the lenticular nucleus on its lateral side and the caudate nucleus and thalamus on its medial aspect. The internal capsule is described as being subdivided into five segments: the anterior limb, genu, posterior limb, retrolentiform part, and sublentiform part (IC-SL). The anterior limb lies between the head of the caudate nucleus medially and the lenticular nucleus laterally; the genu represents the angulated portion between the anterior and posterior limbs; the posterior limb is located between the thalamus medially and the lenticular nucleus laterally; the retrolentiform part is situated posterior to the lenticular nucleus; and the sublentiform part lies inferior to the lenticular nucleus [35].

**Fig. 4:**
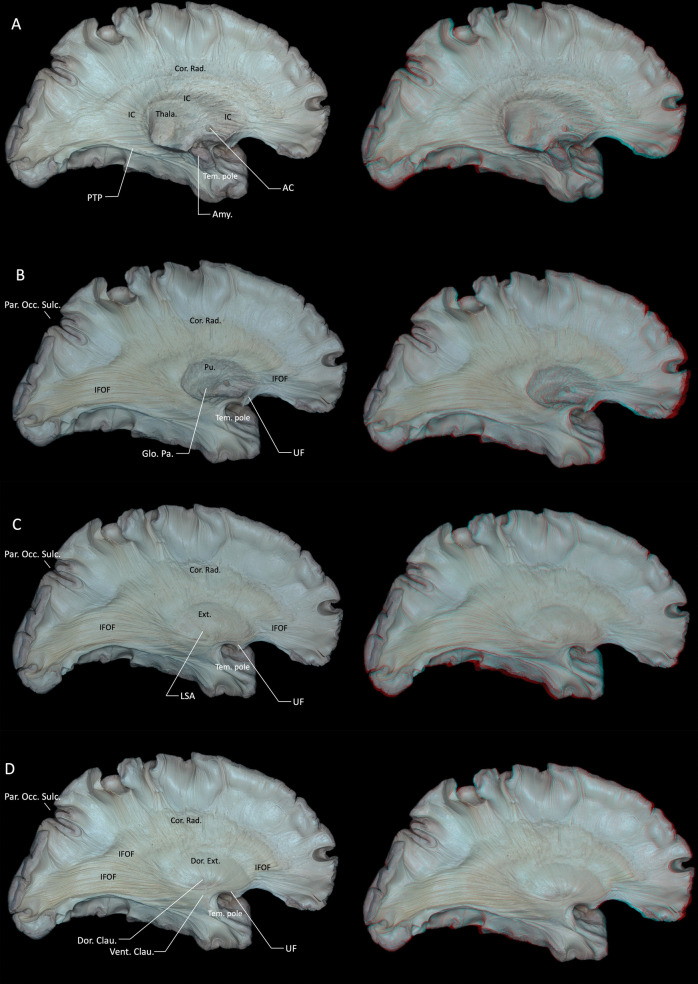
**A** Removing the NAc revealed the entire internal capsule. **B** The thalamic radiations were dissected to expose the fiber tracts located lateral to them, which also expose the IFOF and UF. The UF connects the temporal pole with the orbitofrontal cortex, and the IFOF connects the middle and inferior frontal gyri with the occipital lobe **C** Further dissection of caudate, amygdala and thalamic projection fibers, as well as the lentiform nucleus, exposed the external capsule laterally, demarcated by the LSA separating it from the putamen. **D** The claustrum is a slender sheet of gray matter situated between the extreme capsule laterally and the external capsule medially, which can be divided into dorsal and ventral parts. The dorsal part of the claustrum gives rise to widespread claustrocortical projections that constitute the dorsal external capsule. In contrast, the ventral part of the claustrum lies within the ventral external capsule, which primarily consists of UF and IFOF. AC = anterior commissure; Amy. = amygdala; Clau. = claustrum; CN-H = Head of the caudate nucleus; CN-T = tail of the caudate nucleus; Cor. = corona; Dor. = Dorsal; Ext. = external capsule; IC = internal capsule; IFOF = inferior fronto-occipital fasciculus; LSA = lenticulostriate artery; Occ. = occipital; Par. = parieto; PTP = posterior thalamic peduncle; Rad. = radiata; Tem. = temporal; Thala. = thalamus; UF = uncinate fasciculus; Vent. = ventral. Right-hand panels show 3D reconstructions of the corresponding dissection stages

The IC-SL is located inferior to the lenticular nucleus and superior to the caudate nucleus tail and includes several distinct fiber pathways. These pathways encompass the temporopontine fibers (Türck’s bundle), originating from superior and middle temporal gyri and descending through the posterior limb towards ipsilateral pontine nuclei; occipitopontine fibers originating in the visual cortex of the occipital lobe, following the same trajectory, terminating in the pontine nuclei and subsequently relaying to the contralateral cerebellum via the middle cerebellar peduncle; the inferior thalamic peduncle (ITP), transmitting fibers from the pulvinar of the thalamus to the amygdala and temporal pole; and the posterior thalamic peduncle (PTP), containing reciprocal fibers connecting the pulvinar to temporal and occipital cortices, which include visual and auditory radiations, with optic radiations being a notable component (Fig. 3B) [8, 9, 45]. However, the precise allocation of these tracts to the retrolentiform versus sublentiform parts of the internal capsule remains controversial. Many classical anatomical works describe most of the optic radiation and the posterior thalamic peduncle as belonging predominantly to the retrolentiform part, and the auditory radiation and inferior thalamic peduncle as coursing mainly through the sublentiform part, rather than grouping them together within a single IC-SL compartment [31]. In our specimens, dissection that preserved only the head of the caudate nucleus and removed the stria terminalis exposed fibers of the ITP and PTP along the lateral ventricular roof in the region corresponding to the sublentiform–retrolentiform transition (Fig. 3C). The boundary between the caudate nucleus head and body was identified at the foramen of Monro in the coronal plane (Fig. 3C) [6]. Removal of the caudate head, preserving only the nucleus accumbens (NAc) (Fig. 3D). Removing the NAc revealed the entire internal capsule structure (Fig. 4A). No clear boundary was observed between the nucleus accumbens and the caudate head. Gray matter extensions, known as transcapsular gray matter, connected head and body of the caudate nucleus with the lentiform nucleus. Upon dissecting superficial gray matter from the caudate body and tail, some fiber tracts originating from the superior thalamus entered the caudate nucleus without evident cortical projections (Fig. 3C, D). The thalamic radiations were dissected to expose the fiber tracts located lateral to them (Fig. 4B).

Further dissection of caudate and thalamic projection fibers, together with removal of the lentiform nucleus, exposed the external capsule lateral to the putamen. Lenticulostriate arteries predominantly penetrate the basal ganglia from the anterior perforated substance; only a minority traverse the plane between the putamen and the external capsule. (Fig. 4C) Inferiorly, the inferior fronto-occipital fasciculus (IFOF) and uncinate fasciculus (UF) were identified (Fig. 4C). The UF emerged from the limen insula, crossing the basal frontal region, dividing into anterolateral fibers connecting lateral temporal gyri to te superolateral orbitofrontal cortex and ventromedial fibers connecting the orbital surface, septal area, nucleus accumbens, and medial orbitofrontal region to the amygdala and parahippocampal gyrus (Fig. 4C). The IFOF is a major associative white matter tract that establishes long-range connections between the occipital, parietal, and frontal lobes. The IFOF originates from the medial occipital lobe—particularly the cuneus and lingual gyrus—and potentially from the medial parietal region such as the precuneus. It courses anteromedially through the deep white matter of the temporal lobe, passing deep to the superior and middle temporal gyri and through the temporal stem. It then travels medial to the claustrum and lateral to the putamen, traversing the extreme and external capsules, and ultimately reaches the inferior frontal gyrus (pars opercularis and pars triangularis), the middle frontal gyrus, and the frontal pole (Fig. 4C). The claustrum is a slender sheet of gray matter situated between the extreme capsule laterally and the external capsule medially (Fig. 4D), which can be divided into dorsal and ventral parts. The dorsal part of the claustrum gives rise to widespread claustrocortical projections that constitute the dorsal external capsule. In contrast, the ventral part of the claustrum lies within the ventral external capsule, which primarily consists of UF and IFOF. Superiorly, fibers of the dorsal external capsule ascend and blend with those of the internal capsule above the putamen, collectively forming the corona radiata. Inferiorly, the ventral external capsule is traversed by major association pathways, including the UF and the IFOF. The claustrum appeared throughout the dissection layers, with dorsal claustrum fibers forming a semicircular pattern, and isolated ventral claustrum islands positioned ventrally to the IFOF (Fig. 4D).

**Fig. 5:**
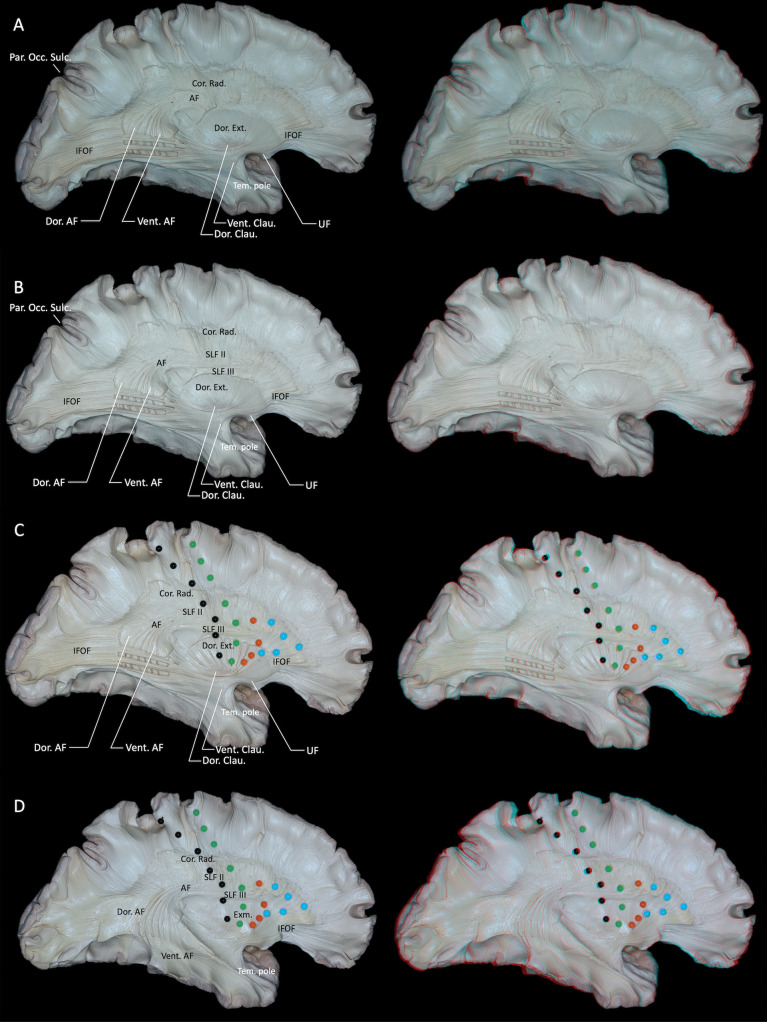
**A** Partial dissection of the IFOF, dorsal external capsule exposed the AF and medial surface of the supramarginal gyrus. **B** The SLF II overlying the AF and the SLF III located beneath it were exposed. **C** The precentral sulcus (green dots), central sulcus (black dots), pars opercularis (orange and green dots), and pars triangularis (blue and green dots) were projected onto the medial surface of the cerebral hemisphere. **D** From the medial surface, the full trajectory of the AF was visualized.The AF is commonly divided into dorsal and ventral segments. The ventral segment projects to frontal areas such as the pars opercularis, pars triangularis, and the ventral premotor cortex. The dorsal segment also targets the pars opercularis and ventral premotor cortex, and in some cases extends to the posterior part of the middle frontal gyrus. Both the dorsal and ventral segments were found to connect to the lateral aspects of the posterior superior, middle, and inferior temporal gyri. Additionally, the ventral component extended along the lateral surface of the entire inferior temporal gyrus and continued anteriorly to reach the temporal pole. AF = arcuate fasciculus; Clau. = claustrum; Cor. = corona; Dor. = Dorsal; Ext. = external capsule; Exm. = extreme capsule; IFOF = inferior fronto-occipital fasciculus; Rad. = radiata; Occ. = occipital; Par. = parieto; SLF = superior longitudinal fasciculus; Tem. = temporal; UF = uncinate fasciculus; Vent. = ventral. Right-hand panels show 3D reconstructions of the corresponding dissection stages

Partial dissection of the IFOF, dorsal external capsule exposed the arcuate fasciculus (AF) and medial surface of the supramarginal gyrus (Fig. 5A). The SLF II overlying the AF and the SLF III located beneath it were exposed (Fig. 5B). Furthermore, the precentral sulcus, central sulcus, pars opercularis, and pars triangularis were projected onto the medial surface of the cerebral hemisphere (Fig. 5C). The AF is commonly divided into dorsal and ventral segments. The ventral segment projects to frontal areas such as the pars opercularis (BA 44), pars triangularis (BA 45), and the ventral premotor cortex (BA 6). The dorsal segment also targets the pars opercularis (BA 44) and ventral premotor cortex (BA 6), and in some cases extends to the posterior part of the middle frontal gyrus. In the present study, both the dorsal and ventral segments were found to connect to the lateral aspects of the posterior superior, middle, and inferior temporal gyri. Additionally, the ventral component extended along the lateral surface of the entire inferior temporal gyrus and continued anteriorly to reach the temporal pole (Fig. 5D) [5, 48].

### Illustrative case: The posterior middle frontal gyrus approach for resection of a high-grade glioma in the right thalamus

**Fig. 6:**
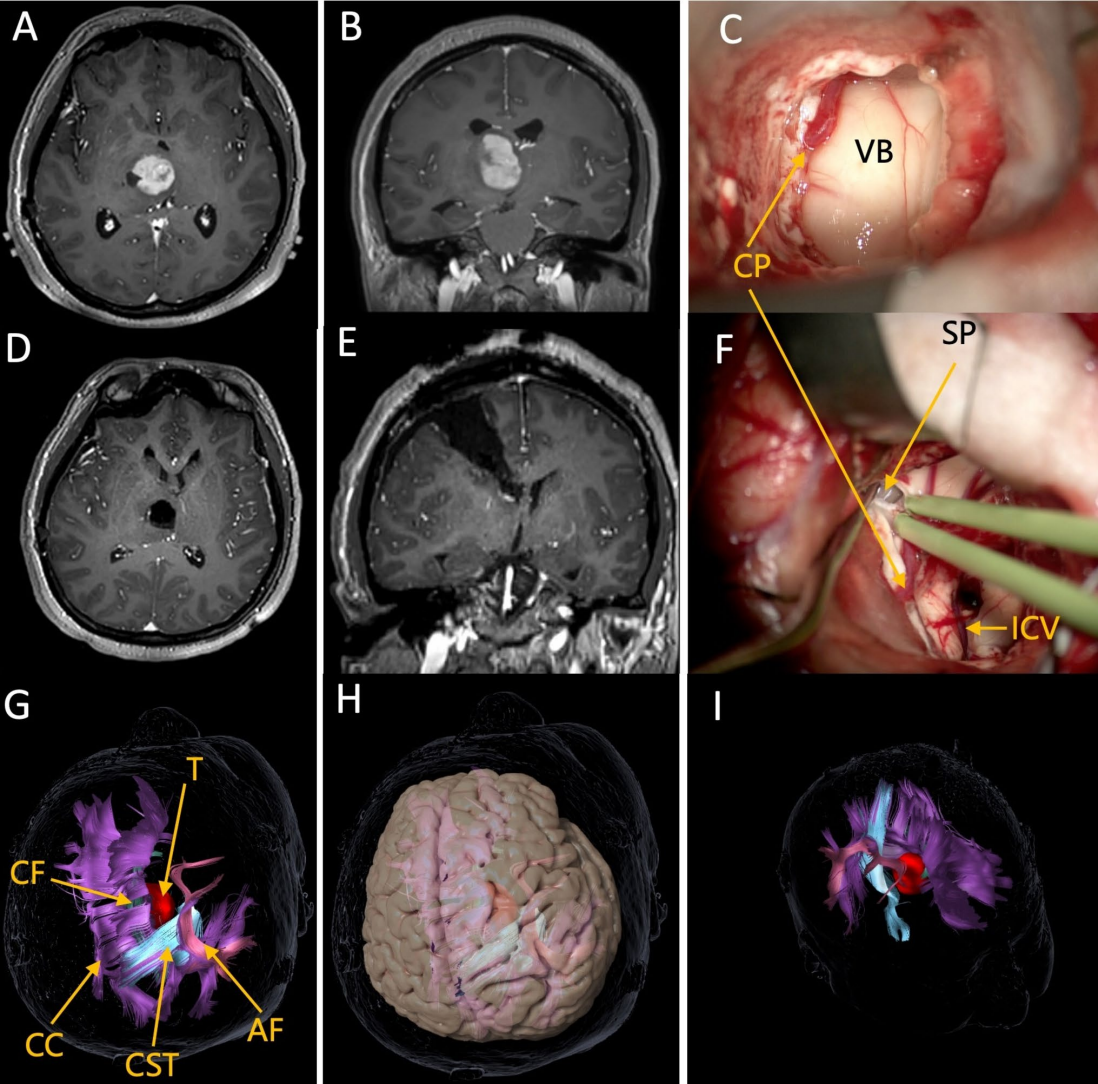
Posterior middle frontal gyrus transcortical–transventricular approach for resection of a right thalamic high-grade glioma. **A** Preoperative axial contrast-enhanced T1-weighted MRI showing an avidly enhancing mass in the medial right thalamus. **B** Preoperative sagittal contrast-enhanced T1-weighted MRI delineating the cranio-caudal extent of the lesion. **C** Intraoperative view (schematic): transcortical entry via the posterior middle frontal gyrus into the body of the lateral ventricle, exposing the superior surface of the thalamus. **D**, **E** Postoperative axial and sagittal contrast-enhanced T1-weighted MRI demonstrating gross-total resection with no residual enhancement. **F** The internal cerebral veins were preserved, and a septostomy of the septum pellucidum was performed. **G** Preoperative imaging of the major white-matter fiber tracts, clearly demonstrating that the surgical corridor avoids critical pathways. **H** A transcortical corticotomy through the middle frontal gyrus can avoid critical white matter fiber tracts. **I** An anterior viewing perspective. AF = arcuate fasciculus; CF = cingulum fasciculus; CC = corpus callosum; CP = choroid plexus; CST = corticospinal tract; ICV = internal cervical vein; SP = Septum pellucidum; T = tumor; VB = ventricular body. Right-hand panels show 3D reconstructions of the corresponding dissection stages

A 54-year-old right-handed woman presented with a 3-month history of intermittent headaches and dizziness. Brain MRI revealed a large, avidly enhancing lesion centered on the medial aspect of the right thalamus (Fig. 6A, B). Because the patient is right-handed and the right hemisphere is nondominant, although the corridor traverses the premotor area and could place the right frontal aslant tract at risk, it was not expected to cause language or motor deficits; moreover, a transcallosal route would risk injury to the corpus callosum and cingulum and traction on cortical draining veins entering the superior sagittal sinus. We therefore selected a posterior middle frontal gyrus transcortical–transventricular approach. Intraoperative neurophysiological monitoring (continuous motor-evoked potentials and direct subcortical stimulation) was used to map and monitor the corticospinal tract (CST). Through a small corticotomy in the posterior middle frontal gyrus, we entered the body of the lateral ventricle, exposed the superior surface of the thalamus (Fig. 6C), incised it, and achieved gross-total resection (Fig. 6D, E). The internal cerebral veins were preserved, and a septostomy of the septum pellucidum was performed (Fig. 6F). Histopathology confirmed glioblastoma, WHO grade 4. The choice of this approach was directly informed by the present anatomical study’s delineation of critical structures along the medial hemisphere and within the central core. Postoperatively, the patient had no neurological deficits, thereby avoiding interhemispheric disconnection syndrome and symptoms related to disruption of the Papez circuit.

## Discussion

Neurosurgeons continuously strive toward the principles articulated by Yasargil, “the more you know, the more you see,” and Rhoton, “gentle, accurate, and safe.” In line with these concepts, this study systematically dissected cerebral white matter from medial to lateral, revealing distinct fiber tracts from a novel internal perspective. A medial-to-lateral dissection trajectory provides a complementary ‘inside-out’ perspective that differs from conventional lateral dissections. Starting from the interhemispheric surface allows sequential exposure of (i) the medial association systems, (ii) commissural systems intimately related to ventricular boundaries (corpus callosum/tapetum), and (iii) projection systems and subcortical nuclei within the central core before reaching lateral association bundles. This workflow helps clarify how these systems are layered, interwoven, and spatially constrained around the ventricles—relationships that are often more difficult to conceptualize from an outside-in approach.

The cingulum comprises long association fibers extending from the paraterminal gyrus, passing superior to the corpus callosum, and reaching the basolateral amygdala, supplemented by shorter fibers from medial frontal cortex, paracentral lobule, precuneus, and cuneus. Functionally, the cingulum serves as the dorsal limbic pathway, critically involved in emotional processing, cognitive functions, attention modulation, and pain perception. It connects crucial limbic structures including the cingulate cortex, parahippocampal region, and entorhinal cortex, integrating emotion and memory with executive control [14, 35]. Fibers from the basolateral amygdala reach the cingulum via two distinct pathways: a rostral pathway through the ventral amygdalofugal tract projecting to the subgenual cingulum and subsequently ascending to the dorsomedial prefrontal areas; and a caudal pathway extending directly to medial temporal structures via the temporal cingulum, bypassing the caudal dorsal cingulum [10]. Our fiber-dissection observations specifically confirm the presence of this caudal amygdala-temporal cingulum connection, highlighting its role in integrating emotional valence with memory consolidation processes. Awareness of this pathway has critical clinical implications in medial temporal lobe surgery, such as epilepsy or tumor resections, where preservation of temporal cingulum integrity is vital to preventing postoperative memory and emotional disturbances.

As the largest commissural tracts, the corpus callosum connects bilateral frontal, parietal, occipital, temporal, insular, and limbic lobes, forming a fundamental anatomical substrate for diverse neurosurgical procedures. In epilepsy surgery, callosotomy effectively interrupts seizure propagation across hemispheres, with microsurgical anatomical studies recommending a transverse incision to minimize functional disruption [16]. In tumor surgery, the corpus callosum, especially its body, serves as a critical route into the third and lateral ventricles. Surgical openings should be restricted to within 3.5 cm posterior to the genu to avoid damaging motor fibers, and splenial injury must be strictly avoided to prevent cognitive deficits such as alexia [28, 38]. Each callosal segment contributes specifically to ventricular boundaries—forceps minor forming the anterior and medial wall of the frontal horn, the body forming the roof of the lateral ventricle body, and the tapetum delineating the lateral walls of atrium, temporal and occipital horns. Resections involving callosal gliomas must carefully consider fiber trajectory; tumors typically infiltrate along specific radiation fibers, underscoring the importance of microsurgical dissection combined with intraoperative imaging and electrophysiological guidance to achieve maximal tumor removal while preserving critical functions.

The fornix, a major hippocampal efferent pathway containing approximately 1.2–2.7 million myelinated fibers per hemisphere, extends from the hippocampal formation to basal forebrain and diencephalon structures [27, 37]. Hippocampal efferents first aggregate at the ventricular floor as the alveus, merge into fimbria, then ascend medially as paired crura beneath the splenium. After merging through the hippocampal commissure, these fibers form the body of the fornix, running horizontally above the thalamus under the septum pellucidum. Rostral bifurcation produces precommissural fibers terminating in basal forebrain nuclei and postcommissural fibers projecting toward anterior thalamic nuclei via mammillary bodies. Functionally critical for declarative and spatial memory, the fornix integrates limbic and autonomic functions [43]. Clinically, fornix integrity is critical in transcallosal and third-ventricle approaches, with injury producing anterograde amnesia; its early atrophy serves as a biomarker in Alzheimer’s disease, and its preservation informs surgical planning in temporal lobe epilepsy and limbic neuromodulation [37]. Studies have shown that bilateral lesions of the anterior fornix columns produce both anterograde and retrograde amnesia, with left-sided damage selectively impairing verbal memory and right-sided damage predominantly affecting spatial memory; overall free recall is markedly reduced while recognition remains relatively spared [3, 25]. In our fiber-tracing study, we further observed that basolateral amygdala projections converge toward the fimbria–fornix complex along the fimbria–body axis, suggesting that amygdalo-hippocampal fibers may join the classical fornix–mammillary–thalamic circuit. This observation is in line with tract-tracing and review studies demonstrating robust amygdalo-hippocampal interconnections and implies that the amygdala can modulate emotional tagging and the encoding of contextual memories via pathways involving the fornix [24]. Nevertheless, given the spatial resolution and methodological limitations of fiber dissection and tractography, this interpretation should be regarded as a structural hypothesis that warrants further experimental validation. Komaitis et al. described a distinct fiber tract known as the fronto-caudate tract (FCT) during dissection of the caudate nucleus, further classifying it into ventral (FCTv) and dorsal (FCTd) segments. The FCTv arises from the frontal pole (BA 10), fronto-orbital cortex (BA 11), ventrolateral prefrontal cortex (BA 47), and ventral anterior cingulate cortex (BA 32), consistently projecting to the caudate head. Conversely, the FCTd originates from the pre-supplementary motor area (BA 6), dorsolateral prefrontal cortex (BA 8), dorsomedial prefrontal cortex (BA 9), and dorsal anterior cingulate cortex (BA 32), projecting medially toward the caudate body via the anterior thalamic radiation. The boundary between these two segments aligns anatomically with the transition from the caudate head to its body near the foramen of Monro, with FCTv notably thicker and more robust than FCTd [15]. Additional diffusion tensor imaging (DTI) and anatomical studies have also explored projections between the caudate nucleus and associated cortical regions [12, 33, 39].

In our dissections, we observed fiber tracts traversing the caudate nucleus, interspersed with patches of gray matter that may represent pathways connecting the caudate to the putamen. These fibers do not appear to originate intrinsically from the caudate itself. We hypothesize that they primarily arise from the thalamus rather than the caudate. Similar fiber-gray matter configurations were also identified in the anterior limb of the internal capsule near the frontal base. Further dissection revealed distinct cortical projections passing through the caudate, suggesting that some internal capsule fibers may traverse the caudate nucleus en route to cortical targets. Although anatomical and MRI studies have demonstrated gray-matter connections between the caudate nucleus and the putamen, to our knowledge there are still no reports describing fiber tracts originating from the thalamus that project to the head and body of the caudate nucleus [11, 20, 32].

Although previous studies indicate that the IFOF does not have a direct anatomical counterpart in macaque monkeys, it may correspond to the extreme capsule in these primates. The IFOF has been described as connecting the inferomedial occipital and possibly medial parietal cortices (precuneus) with the inferior frontal gyrus, mid-portion of the middle frontal gyrus (dorsolateral prefrontal cortex), and frontal pole via the superior and middle temporal gyri and temporal stem [4]. In our study, we further demonstrated that the IFOF also consistently projects to the posterior aspect of the parietal lobe. Functionally, the IFOF is associated with semantic processing, visual recognition, integration of multimodal sensory input and motor planning, as well as reading, writing, and language comprehension [6, 34]. Intraoperative electrostimulation of the IFOF has been reported to elicit semantic paraphasias, indicating its critical role in semantic processing [6]. It also serves as the medial boundary for left temporal lobectomy procedures, helping surgeons avoid postoperative language deficits. Furthermore, the IFOF covers the optic radiations as they travel deep to the superior and middle temporal gyri, occipital lobe, and adjacent to the lateral ventricle's temporal horn, atrium, and occipital horn. Precise surgical techniques to avoid penetration into deep IFOF layers aid in preserving visual functions. Given its complex functional roles in language, theory of mind, reading, attention, emotional processing, and motor cognition, the IFOF likely interacts with numerous other function-specific fiber tracts.

Mirand et al. and Kaan et al. have proposed that the claustrum and external capsule are subdivided into dorsal and ventral segments. The dorsal external capsule primarily contains claustrocortical fibers converging into dorsal claustrum gray matter. The ventral external capsule comprises fibers from the uncinate fasciculus and IFOF traversing the ventral claustrum, connecting the orbitofrontal and prefrontal cortices to amygdaloid, temporal, and occipital areas [7]. Despite previous suggestions that the claustrum and IFOF are anatomically separate, with the IFOF positioned ventrally, the precise boundary between these two structures remains unclear. Martino and colleagues have further refined the organization of the IFOF into dual layers: a superficial (ventral) layer connecting inferior frontal regions (BA 45, 47) with inferior occipital, inferior temporal, and fusiform cortices (BA 19, 37); and a deeper (dorsal) layer linking orbitofrontal and superior/middle frontal regions (BA 8, 9, 10, 11) to superior/middle occipital and superior parietal cortices (BA 7, 17, 18) [23, 34].

Previous studies using fiber dissection and diffusion tensor imaging (DTI) have described two main segments of the AF. The dorsal segment originates from the posterior superior temporal gyrus (BA 22, 41, 42) and the mid-portion of the middle temporal gyrus (BA 21), then courses slightly ventral to the superior longitudinal fasciculus II (SLF II) to reach the inferior and middle frontal gyri. The ventral segment, on the other hand, arises from the posterior middle temporal gyrus (BA 37) and, less frequently, from the inferior temporal gyrus (BA 20). It ascends through the lower part of the supramarginal gyrus and passes medial to the SLF III within the frontoparietal operculum, terminating in the inferior frontal gyrus [45, 47, 48, 51]. However, in our study, we found that when examined from a medial-to-lateral perspective, the AF exhibits a more complex pattern of connectivity within the temporal lobe.

The AF is bilateral but functionally asymmetric. In most right-handed individuals, the left AF is dominant for language and supports phonological encoding, speech repetition, syntactic processing, and verbal fluency; lesions typically produce conduction aphasia [48]. By contrast, the right AF—while less critical for core language—contributes to prosody and pitch-based aspects of speech and music, auditory–motor integration, and elements of working memory and motor planning. Accordingly, AF-related functions engage both hemispheres, with left-hemisphere predominance for language and right-hemisphere contributions to prosody and non-linguistic auditory functions [48]. This study further complements and refines the understanding of the structural continuity and extent of the AF.

## Limitations

This study represents a qualitative, layer-by-layer anatomical investigation of the cerebral hemisphere performed from a medial-to-lateral perspective using fiber dissection. The number of specimens was limited, and no quantitative morphometric data were collected. Validation through additional modalities, including diffusion imaging or neuronal tracing in animal models, is necessary to confirm the presence and functional relevance of this connection. Future integration of anatomical dissection with advanced imaging may facilitate the generation of high-fidelity 3D reconstructions for research and surgical planning.

## Conclusion

This study provides a comprehensive dissection of the cerebral hemisphere from medial to lateral, revealing the sequential architecture of white matter pathways and subcortical structures. Compared to imaging alone, 3D anatomical visualization offers a more intuitive understanding of spatial relationships, particularly for deep-seated regions that are difficult to access surgically. By correlating internal fiber anatomy with surface landmarks, this approach may aid neurosurgeons—especially those early in training—in navigating complex basal brain regions more safely and accurately. These findings contribute to the refinement of minimally invasive neurosurgical approaches and enhance our understanding of deep brain connectivity from a novel anatomical perspective.

## Supplementary Information

Below is the link to the electronic supplementary material.

ESM 1 Supplementary Material 1 (MOV 244 MB)

## Data Availability

The data analyzed in this study are provided directly in the figures.
